# Body Mass Index and Diabetes in Asia: A Cross-Sectional Pooled Analysis of 900,000 Individuals in the Asia Cohort Consortium

**DOI:** 10.1371/journal.pone.0019930

**Published:** 2011-06-22

**Authors:** Paolo Boffetta, Dale McLerran, Yu Chen, Manami Inoue, Rashmi Sinha, Jiang He, Prakash Chandra Gupta, Shoichiro Tsugane, Fujiko Irie, Akiko Tamakoshi, Yu-Tang Gao, Xiao-Ou Shu, Renwei Wang, Ichiro Tsuji, Shinichi Kuriyama, Keitaro Matsuo, Hiroshi Satoh, Chien-Jen Chen, Jian-Min Yuan, Keun-Young Yoo, Habibul Ahsan, Wen-Harn Pan, Dongfeng Gu, Mangesh Suryakant Pednekar, Shizuka Sasazuki, Toshimi Sairenchi, Gong Yang, Yong-Bing Xiang, Masato Nagai, Hideo Tanaka, Yoshikazu Nishino, San-Lin You, Woon-Puay Koh, Sue K. Park, Chen-Yang Shen, Mark Thornquist, Daehee Kang, Betsy Rolland, Ziding Feng, Wei Zheng, John D. Potter

**Affiliations:** 1 Mount Sinai School of Medicine, The Tisch Cancer Institute, New York, New York, United States of America; 2 International Prevention Research Institute, Lyon, France; 3 Department of Public Health Sciences, Fred Hutchinson Cancer Research Center, Seattle, Washington, United States of America; 4 Department of Environmental Medicine, New York University School of Medicine, New York, New York, United States of America; 5 Epidemiology and Prevention Division, Research Center for Cancer Prevention and Screening, National Cancer Center, Tokyo, Japan; 6 Division of Cancer Epidemiology and Genetics, National Cancer Institute, Bethesda, Maryland, United States of America; 7 Department of Epidemiology, Tulane University School of Public Health and Tropical Medicine, New Orleans, Louisiana, United States of America; 8 Healis-Sekhsaria Institute for Public Health, Navi Mumbai, India; 9 Department of Health and Social Services, Ibaraki Prefectural Government, Ibaraki, Japan; 10 Department of Public Health, Aichi Medical University School of Medicine, Aichi, Japan; 11 Department of Epidemiology, Shanghai Cancer Institute, Shanghai, China; 12 Division of Epidemiology, Department of Medicine, Vanderbilt Epidemiology Center, Vanderbilt University, Nashville, Tennessee, United States of America; 13 Masonic Cancer Center, University of Minnesota, Minneapolis, Minnesota, United States of America; 14 Division of Epidemiology, Department of Public Health and Forensic Medicine, Tohoku University Graduate School of Medicine, Sendai, Japan; 15 Division of Epidemiology and Prevention, Aichi Cancer Center Research Institute, Nagoya, Japan; 16 Environmental Health Sciences, Tohoku University Graduate School of Medicine, Sendai, Japan; 17 Genomics Research Center, Academia Sinica, Taipei, Taiwan; 18 Graduate Institute of Epidemiology, College of Public Health, National Taiwan University, Taipei, Taiwan; 19 Department of Preventive Medicine, Seoul National University College of Medicine, Seoul, South Korea; 20 Departments of Health Studies, Medicine and Human Genetics and Cancer Research Center, The University of Chicago, Chicago, Illinois, United States of America; 21 Institute of Biomedical Sciences, Academia Sinica, Taipei, Taiwan; 22 Department of Biochemical Science and Technology, National Taiwan University, Taipei, Taiwan; 23 Fuwai Hospital and Cardiovascular Institute, Chinese Academy of Medical Sciences and Peking Union Medical College, China National Center for Cardiovascular Disease, Beijing, China; 24 Department of Public Health, Dokkyo Medical University School of Medicine, Tochigi, Japan; 25 Division of Epidemiology, Miyagi Cancer Center Research Institute, Miyagi, Japan; 26 Department of Epidemiology and Public Health, Yong Loo Lin School of Medicine, National University of Singapore, Singapore, Singapore; 27 Department of Preventive Medicine, Seoul National University College of Medicine and Cancer Research Institute and Institute of Health Policy and Management, Seoul National University, Seoul, South Korea; 28 Taiwan Biobank, Institute of Biomedical Sciences, Academia Sinica, Taipei, Taiwan; 29 Graduate Institute of Environmental Science, China Medical University, Taichung, Taiwan; National Institutes of Health - National Institute of Child Health and Human Development, United States of America

## Abstract

**Background:**

The occurrence of diabetes has greatly increased in low- and middle-income countries, particularly in Asia, as has the prevalence of overweight and obesity; in European-derived populations, overweight and obesity are established causes of diabetes. The shape of the association of overweight and obesity with diabetes risk and its overall impact have not been adequately studied in Asia.

**Methods and Findings:**

A pooled cross-sectional analysis was conducted to evaluate the association between baseline body mass index (BMI, measured as weight in kg divided by the square of height in m) and self-reported diabetes status in over 900,000 individuals recruited in 18 cohorts from Bangladesh, China, India, Japan, Korea, Singapore and Taiwan. Logistic regression models were fitted to calculate cohort-specific odds ratios (OR) of diabetes for categories of increasing BMI, after adjustment for potential confounding factors. OR were pooled across cohorts using a random-effects meta-analysis. The sex- and age-adjusted prevalence of diabetes was 4.3% in the overall population, ranging from 0.5% to 8.2% across participating cohorts. Using the category 22.5–24.9 Kg/m^2^ as reference, the OR for diabetes spanned from 0.58 (95% confidence interval [CI] 0.31, 0.76) for BMI lower than 15.0 kg/m^2^ to 2.23 (95% CI 1.86, 2.67) for BMI higher than 34.9 kg/m^2^. The positive association between BMI and diabetes prevalence was present in all cohorts and in all subgroups of the study population, although the association was stronger in individuals below age 50 at baseline (p-value of interaction<0.001), in cohorts from India and Bangladesh (p<0.001), in individuals with low education (p-value 0.02), and in smokers (p-value 0.03); no differences were observed by gender, urban residence, or alcohol drinking.

**Conclusions:**

This study estimated the shape and the strength of the association between BMI and prevalence of diabetes in Asian populations and identified patterns of the association by age, country, and other risk factors for diabetes.

## Introduction

The prevalence of diabetes has increased in the last decades in many low- and middle-income countries (LMIC) [1] and this trend, which is almost completely accounted for by type 2 diabetes, is expected to continue [2]. The current estimated age-adjusted prevalence of diabetes in China (4.2%) is expected to increase by 1.9% to 5.0% in 2030 [3]. However, results from a survey conducted in 2007-08 in China reported a higher prevalence (9.7%, including previously undiagnosed diabetes) [4]. A similar increase is expected in India (from 7.8% in 2010 to 9.3% in 2030) and in high-prevalence countries such as Malaysia (from 11.6% in 2010 to 13.8% in 2030) [3]. Southern and Eastern Asian countries account for half the deaths attributable to diabetes worldwide [1], [5] and the consequences of a rising incidence and prevalence of diabetes and other chronic diseases will be particularly important, both locally and globally [5]. A concurrent increase in the prevalence of overweight and obesity over the same time period has been observed in many Asian countries [6]. In rural China, the prevalence of overweight has increased from 5.3% in men and 9.8% in women in 1992 [7] to 13.6% in men and 14.4% in women in 2002 [8]. Corresponding figures for obesity were 0.5% (men) and 0.7% (women) in 1992 and 1.8% (men) and 3.0% (women) in 2002. This trend has profound implications for the expected number of diabetes patients who will be diagnosed in this region in future decades.

Lifestyle risk factors for type 2 diabetes include increased body weight, weight gain, and lack of physical activity [9]. A causal association with tobacco smoking is suspected [10]. Roles for specific foods such as fruits, vegetables and low-fat dairy, and nutrients, including unsaturated fat and fibre, in reducing diabetes risk, are suspected but not established [11]. Increased body mass is one of best investigated risk factors for the disease, but the evidence comes primarily from studies conducted in populations of European origin. With few exceptions [12]–[14], studies on the relation between body mass and risk of type 2 diabetes in Asia have been conducted among convenience, small-scale samples. The proportion of healthy adults with a body mass lower than 20 kg/m^2^ is larger in Asia than in high-income countries; the greater population variation in Asians may offer an opportunity to better characterize the role of BMI in determining diabetes risk.

The Asia Cohort Consortium (ACC) is a collaboration of longitudinal studies aimed at sharing resources to investigate the distribution and determinants of chronic diseases across the continent [15]. The Consortium provided the opportunity to study the relation between BMI and prevalence of diabetes in a large population across several countries of South and East Asia.

## Materials and Methods

### Ethics Statement

This study was approved by the Institutional Review Board of the Fred Hutchinson Cancer Research Center. Informed consent was obtained by individual studies.

### Study Population and Data Collection

The cohorts included in this project were identified through a systematic literature search, followed by a survey that was sent to the investigators of each cohort to further determine study eligibility. Eighteen cohorts were included in the pooling project. Approval has been obtained from relevant ethics committees.

All participating cohorts were required to have data on diabetes at baseline (self-reported), BMI at baseline (measured or self-reported), age, and sex. Additional data were available from selected cohorts on tobacco smoking (18 cohorts), alcohol drinking (14 cohorts), education (10 cohorts), and urban/rural residence (18 cohorts). Data on waist measurement were available only from a small subset of the whole study population and were not used in the analysis. Information on type of diabetes and age at diagnosis was not available.

Individual data from participating cohorts were harmonized and analyzed at the ACC coordinating center at the Fred Hutchinson Cancer Research Center. This harmonization process involved several rounds of discussions with each cohort to ensure that variables had been correctly interpreted and extracted. Cohort-specific analyses were distributed to each cohort's data management team for review to ensure that the results were in line with the cohort's own analyses.

Repeated measurements of BMI during follow-up were available from five cohorts, for a total of 121,732 individuals, of whom 37,399 had one repeated measurement, 72,531 two repeated measurements and 11,802 three. The yearly change in mean BMI was compared between diabetic and non-diabetic individuals.

### Statistical Analysis

After exclusion of subjects with missing data on diabetes (n = 79,414), age (n = 2), or BMI (n = 11,696), 934,154 subjects were included in the analysis (452,785 men, 481,369 women). The prevalence of diabetes was adjusted by age and sex using direct standardization, based on the sex and age distribution of the whole study population. The association between diabetes and BMI was examined in logistic regression models employing a categorical representation of BMI as the independent variable. Ten BMI levels were established: <15.0, 15.0–17.4, 17.5–19.9, 20.0–22.4, 22.5–24.9, 25.0–27.4, 27.5–29.9, 30.0–32.4, 32.5–34.9, and ≥35.0 kg/m^2^. The categories were chosen to increase our ability to investigate the association between BMI and diabetes, in particular at the extremes of the BMI distribution. We used the standard cut-points for underweight, overweight and obesity rather than those proposed by the World Health Organization for Asian populations [16] to enable direct comparisons with studies conducted in other populations. However, we conducted additional analyses using the ‘Asian’ cut-points (<18.5, 18.5–22.9, 23.0–24.9, 25.0–29.9, ≥30.0). Using the BMI range 22.5–25.0 kg/m^2^ as the reference category (23.0–24.9 in the analysis with ‘Asian’ cut-points), odds ratios (OR) and 95% confidence intervals (CI) were estimated for the other BMI categories, after adjusting for probable confounders, including baseline age ( <40, 40–49, 50–59, 60–69, 70–79, and ≥80) and sex. Additional variables were adjusted for in some analyses, namely tobacco smoking (ever/never), alcohol drinking (ever/never), education (low [less than secondary school]; medium [secondary school]; high [more than secondary school]), and area of residence (urban or suburban; rural).

In analyses presented here, the association between BMI and diabetes prevalence was estimated separately for each cohort. We assumed that the cohort-specific log OR for BMI has a fixed-effect component that is common to all cohorts and a random effect that is cohort-specific. The random effects for the log OR were assumed to be normally distributed, with mean zero; that is, we assumed that 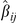, the estimated log OR for the j-th BMI level in the i-th cohort, has distribution 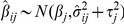 where 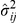 is the within-study variance of 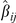 as estimated from the logistic regression model [17], [18]. Parameters 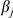 and 95% confidence intervals were estimated in the meta-analysis. The random effects model employs a weighting scheme based on the inverse of the variance; in general, weights are closely approximated by cohort size, but also depend on the between cohort variance. Linear trends were analyzed by fitting logistic regression models including BMI as a continuous variable: in these analyses, the unit of exposure is one kg/m^2^. Logistic regression analysis was performed using the GENMOD procedure in SAS version 9.2 [19]. The meta-analysis was performed using the SAS MIXED procedure [19].

In order to explore possible heterogeneity by Asian ethnicity and economic development, analyses were conducted agglomerating cohorts into three regions: (i) India and Bangladesh; (ii) China, Taiwan, Korea, and Singapore; (iii) Japan. Additional stratified analyses were also performed by sex, age groups, education, tobacco smoking, and alcohol drinking. In stratified analyses, a cohort- and stratum-specific logistic regression model was fit with BMI categories as a predictor. For each BMI level, effects of the stratification factor were examined in a meta-analysis regressing log OR (relative to the BMI reference level) against the stratification factor entered as a fixed effect while allowing for cohort random effects. Stratum-specific least squares mean values of the log OR are reported. Assumptions about stratum-specific differential change in diabetes prevalence across the entire BMI range were examined in a meta-analysis of BMI effect estimates where BMI had been included in the first stage as a continuous linear predictor. More highly stratified analyses in which differential effects of sex, age, and smoking status across the three Asian subgroups were examined in a similar manner, but with the addition of tests of the interaction between subgroup by stratification variable obtained in the meta-analysis. In sensitivity analyses, we have excluded three cohorts which were established in the 1980s.

## Results

Table 1 shows the key characteristics of cohorts included in the analysis. The size of the studies ranged from 5,000 individuals in one study from Taiwan to about 150,000 in one study from China. Enrolment in the studies ranged from the mid-1980s (one study from China and two from Japan - these cohorts were excluded in a sensitivity analysis) to the early 2000's (studies from Bangladesh, Korea and one from China): the majority of subjects were enrolled in the 1990s. The average age at baseline was 54.5 years (standard deviation [sd] 10.4; range of cohort-specific averages 37.1-60.5). Nine of the cohorts had self-reported information on BMI, while height and weight were measured in the other nine cohorts; the average BMI was 23.5 kg/m^2^ (sd 3.5; range 19.8 – 24.0). Overall, 39,794 cohort members (adjusted prevalence 4.3%) reported diabetes at baseline: the age- and sex-adjusted prevalence was lowest in one study from China (0.5%) and highest in the study from Singapore (8.2%). Table 1 also reports information on cohort members with missing information on diabetes: the proportion of members with missing information was above 10% in three cohorts, between 1 and 10% in three additional cohorts, and below 1% in the remaining 12 cohorts. With the exception of the cohort from India, subjects with missing information were older than subjects with valid information; the mean BMI, however, was similar in subjects with and without missing information.

**Table 1:** Characteristics of the cohorts included in the pooled analysis.

		Cohort members with non-missing information on diabetes							Diabetes missing	
		No of	Period of	Age at		Diabetes at	Urban	%	No of	
Cohort	Ref*	subjects^a^	enrolment	baseline^b^	BMI b	baseline (%)^c^	(%)	males	subjects^a^	Percent
Japan										
3 Pref Aichi	[a1]	22,641	1985	54.6	22.1 (2.9)	4.8	100.0	47.2	10,405	46.0
Ibaraki	[a2]	97,608	1993–1994	58.8	23.5 (3.2)^d^	2.5	100.0	31.2	0	0.0
JACC	[a3]	75,486	1988–1990	57.0	22.8 (3.3)	4.9	100.0	42.0	11,196	14.8
JPHC1	[a4]	42,771	1990	49.6	23.6 (3.0)	2.5	100.0	47.8	0	0.0
JPHC2	[a4]	55,712	1993–1994	54.2	23.5 (3.1)	4.9	100.0	47.2	0	0.0
3 Pref Miyagi	[a1]	29,554	1984	56.9	23.3 (3.4)	5.5	54.4	45.0	0	0.0
Miyagi	[a5]	44,868	1990	52.0	23.6 (3.0)	3.9	100.0	47.9	0	0.0
Ohsaki	[a6]	47,710	1995	60.1	23.5 (3.3)	5.3	100.0	48.2	0	0.0
China										
CHEFS	[a7]	143,884	1990–1992	55.2	22.6 (3.7)^d^	2.1	59.4	49.2	10,907	7.6
SCS^e^	[a8]	18,100	1986–1989	55.3	22.2 (3.0)	0.5	100.0	100.0	0	0.0
SMHS^e^	[a9]	61,379	2001–2006	54.9	23.7 (3.1)^d^	3.0	100.0	100.0	0	0.0
SWHS^f^	[a10]	74,881	1996–2000	52.1	24.0 (3.4)^d^	2.7	100.0	0.0	0	0.0
Taiwan										
CBCSP	[a11]	23,703	1991–1992	47.3	24.0 (3.4)^d^	3.1	64.6	50.3	65	0.3
CVDFACTS	[a12]	5,128	1990–1993	47.0	23.7 (3.5)^d^	5.0	100.0	44.1	3	0.1
Korea										
KMCC	[a13]	15,058	1993–2004	55.5	23.6 (3.2)^d^	4.7	58.4	39.8	955	6.3
Singapore										
SCHS	[a14]	63,257	1993–1998	56.5	23.1 (3.3)	8.2	100.0	44.2	0	0.0
Bangladesh										
HEALS	[a15]	11,149	2000–2002	37.0	19.8 (3.2)^d^	5.3	0.0	42.5	321	2,9
India										
MCS	[a16]	101,265	1991–1997	51.8	22.2 (4.1)^d^	2.5	100.0	68.3	45,562	45.0
Total		934,154	1984–2006	54.5	23.1 (3.5)	4.3	89.5	48.5	79,414	8.5

*See Reference List S1

a Minor differences in the number of subjects compared to the original publications are explained by selection criteria in the different analyses

b Mean (SD) for BMI and mean for age at baseline.

c Prevalence of diabetes is standardized for age and sex.

d BMI was estimated using weight and height measured at enrollment. For other studies weight and height were self-reported.

e Cohort restricted to men.

f Cohort restricted to women.

There was a six-fold difference in the age- and sex-adjusted prevalence of diabetes between the category with highest BMI (35.0 kg/m^2^ or higher; prevalence 8.6%) and that with the lowest BMI (less than 15 kg/m^2^; prevalence 1.3%). The prevalence OR for diabetes in relation to BMI categories ranged from 0.58 (95% CI 0.31, 0.76) to 2.23 (95% CI 1.86, 2.67) (Table 2). The prevalence of diabetes increased linearly across the range of BMI categories, with an overall coefficient equal to 0.084/Kg/m^2^ (i.e., the OR of diabetes for an increase in one Kg/m^2^ BMI was 1.088). The cohort-specific coefficients ranged from 0.036 to 0.200. Adjustment for additional covariates, including education, urban residence, and tobacco smoking, did not alter the results presented in Table 2 (not reported in detail).

**Table 2:** Odds ratios of diabetes for body mass index, overall and stratified by sex and age.

Body mass index at baseline (Kg/m^2^)											
	<15.0	15.0–17.4	17.5–19.9	20.0–22.4	22.5–24.9	25.0–27.4	27.5–29.9	30.0–32.4	32.5–34.9	35.0–50.0	Slope (SE)
**All subjects (n = 934,154)** a											
N of cases (prevalence %)	59 (1.4)	530 (1.7)	3309 (2.6)	8795 (3.5)	12743 (4.7)	8279 (5.3)	3934 (6.3)	1464 (7.2)	427 (8.0)	254 (8.8)	
OR a	0.58	0.49	0.57	0.76	1.00	1.21	1.46	1.74	2.05	2.23	0.084
(95% CI)	(0.31,0.76)	(0.35,0.68)	(0.46,0.72)	(0.67,0.86)	(reference)	(1.13,1.29)	(1.33,1.61)	(1.51,2.00)	(1.75,2.40)	(1.86,2.67)	(0.010)
**Men (n = 452,785)**											
N of cases (prevalence %)	34 (1.7)	289 (1.9)	1936 (3.0)	5306 (4.2)	7257 (5.4)	4519 (6.1)	1896 (7.4)	577 (8.0)	142 (9.0)	67 (9.0)	
OR a	0.65	0.52	0.57	0.75	1.00	1.21	1.45	1.71	2.04	1.93	0.084
(95% CI)	(0.35,1.21)	(0.35,0.77)	(0.44,0.74)	(0.65,0.87)	(reference)	(1.12,1.31)	(1.20,1.64)	(1.44,2.03)	(1.67,2.49)	(1.49,2.50)	(0.013)
**Women (n = 481,369)**											
N of cases (prevalence %)s	25 (1.2)	241 (1.6)	1373 (2.1)	3489 (2.8)	5486 (3.9)	3760 (4.6)	2038 (5.6)	887 (6.8)	285 (7.6)	187 (8.7)	
OR a	0.47	0.47	0.59	0.79	1.00	1.24	1.50	1.78	2.13	2.42	0.081
(95% CI)	(0.31,0.71)	(0.36,0.62)	(0.48,0.71)	(0.71,0.87)	(reference)	(1.13,1.35)	(1.35,1.66)	(1.52,2.09)	(1.77,2.57)	(1.89,3.10)	(0.008)
											
**Age<50 (n = 334,515)**											
N of cases (prevalence %)	6 (0.5)	59 (0.6)	468 (1.0)	1271 (1.3)	1877 (2.0)	1386 (2.6)	696 (3.5)	294 (4.6)	84 (4.9)	41 (4.8)	
OR a	2.55	0.71	0.58	0.69	1.00	1.36	1.90	2.77	3.44	3.95	0.117*
(95% CI)	(0.66,9.83)	(0.45,1.13)	(0.46,0.72)	(0.60,0.78)	(reference)	(1.21,1.53)	(1.62,2.24)	(2.28,3.36)	(2.57,4.61)	(2.85,5.47)	(0.010)
**Age 50–59 (n = 292,225)**											
N of cases (prevalence %)	11 (1.2)	130 (1.7)	975 (2.7)	2738 (3.6)	4142 (4.6)	2776 (5.3)	1275 (6.1)	502 (7.3)	148 (8.4)	89 (9.6)	
OR a	0.75	0.67	0.64	0.76	1.00	1.21	1.42	1.76	2.29	2.76	0.082*
(95% CI)	(0.29,1.95)	(0.40,1.12)	(0.49,0.84)	(0.66,0.89)	(reference)	(1.12,1.31)	(1.27,1.59)	(1.49,2.09)	(1.87,2.80)	(2.21,3.46)	(0.013)
**Age≥60 (n = 307,414)**											
N of cases (prevalence %)	42 (2.0)	341 (2.6)	1866 (4.2)	4786 (6.1)	6724 (7.7)	4117 (8.1)	1963 (9.2)	668 (9.6)	195 (10.6)	124 (11.2)	
OR a	0.59	0.46	0.55	0.80	1.00	1.15	1.32	1.39	1.68	1.79	0.076*
(95% CI)	(0.29,1.19)	(0.29,0.71)	(0.41,0.72)	(0.70,0.91)	(reference)	(1.07,1.24)	(1.19,1.47)	(1.17,1.67)	(1.40,2.01)	(1.48,2.16)	(0.011)

aMeta-analysis estimates of cohort-specific OR adjusted for age and sex (when appropriate).

*P-value of difference across strata <0.001.

OR, odds ratio; CI, confidence interval; SE, standard error.

Women reported a lower prevalence of diabetes than men (age-adjusted prevalence, 3.7% vs. 4.8%, p-value <0.0001), but the shape of the association between BMI and prevalence of diabetes was consistent between the sexes (p-value for interaction 0.8). The prevalence of diabetes strongly increased with age (sex-adjusted prevalence 1.9% below age 50, 4.4% in the age group 50–59, and 6.8% at age 60 or above); although, in the low-BMI range, the OR for diabetes were comparable in these three age groups, the association between high BMI and diabetes prevalence was stronger in younger than in older individuals (p-value for interaction <0.0001). Specifically, ORs in the categories 27.5–29.9, 30.0–32.4, 32.5–35.0 and more than 35.0 kg/m^2^ were 1.90, 2.77, 3.44 and 3.95 below age 50, 1.42, 1.76, 2.29 and 2.76 in the age group 50–59, and 1.32, 1.39, 1.68, 1.79 at age 60 and above.

**Figure 1:**
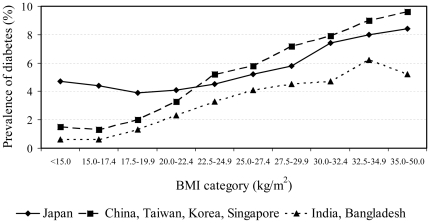
Sex- and age-adjusted prevalence of diabetes by geographic region.

**Figure 2:**
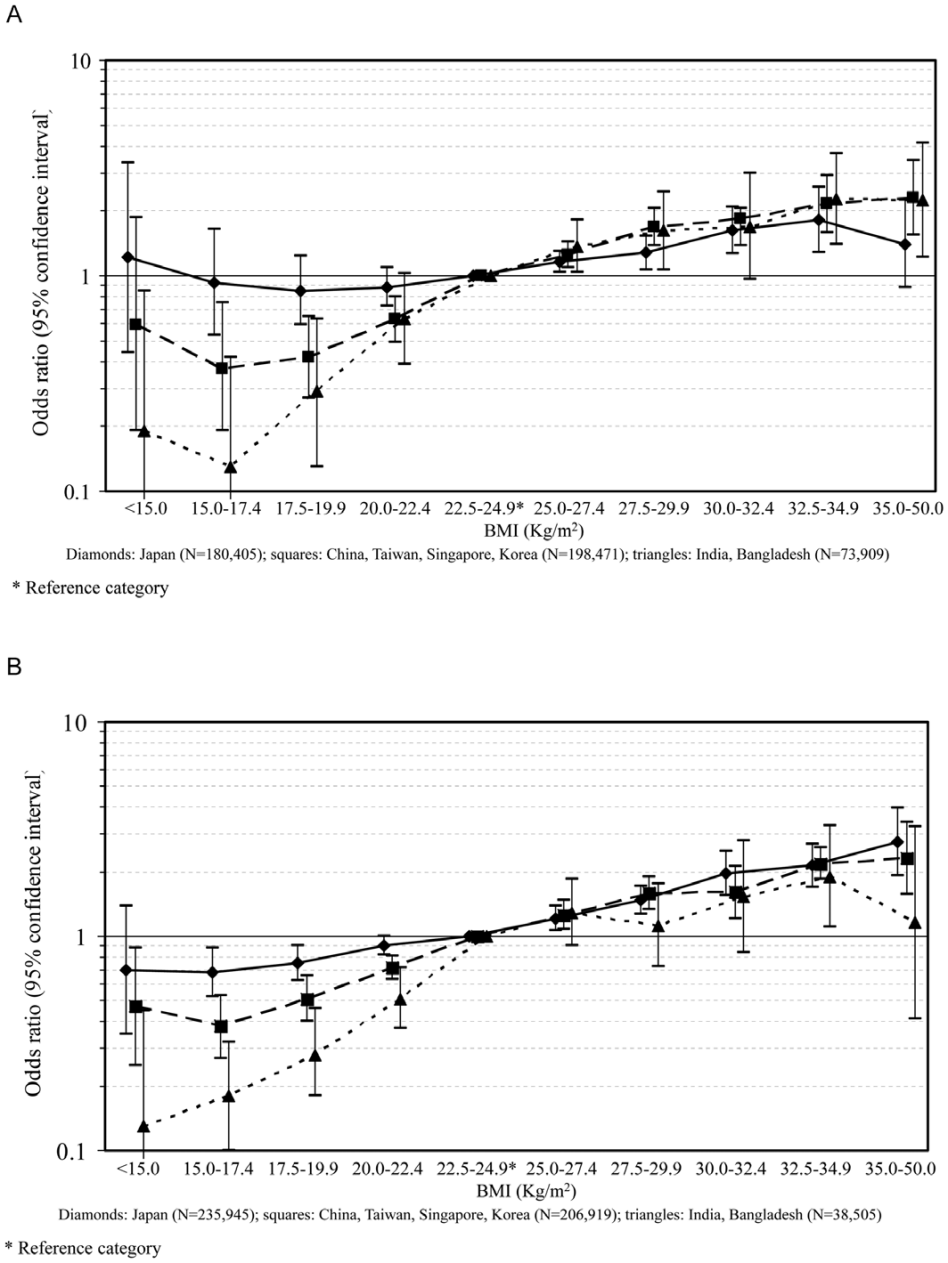
A. OR of diabetes for BMI category in men, stratified by geographic region. B. OR of diabetes for BMI category in women, stratified by geographic region.

Figure 1 shows the age- and sex-adjusted prevalence of diabetes in categories of BMI in the three geographic regions (Japan; China, Taiwan, Korea, and Singapore; India and Bangladesh), and Figures 2A and 2B show the corresponding ORs for men and women, respectively. The prevalence of diabetes was consistently lower in India and Bangladesh than in the other countries (Figure 1). Compared with China, Taiwan, Korea, and Singapore, the prevalence in Japan was higher among individuals with BMI lower than 22.5 kg/m^2^; in individuals with higher body mass, however, the prevalence of diabetes in the two regions was similar (Figure 1).

Among both men and women, the apparent protection exerted by low body mass (below 22.5 kg/m^2^) on diabetes prevalence was more pronounced in India and Bangladesh than in the other countries included in the study (Figures 2A and 2B). In China, Taiwan, Korea, and Singapore this effect was nonetheless stronger than in Japan. Conversely, for high BMI (above 25 kg/m^2^), no clear differences emerged among the country-specific results. A similar pattern was seen from the separate analysis of each individual cohort, although the numbers of cases of diabetes included in some categories were rather small, thus limiting the precision of the results (Table S1).

**Figure 3:**
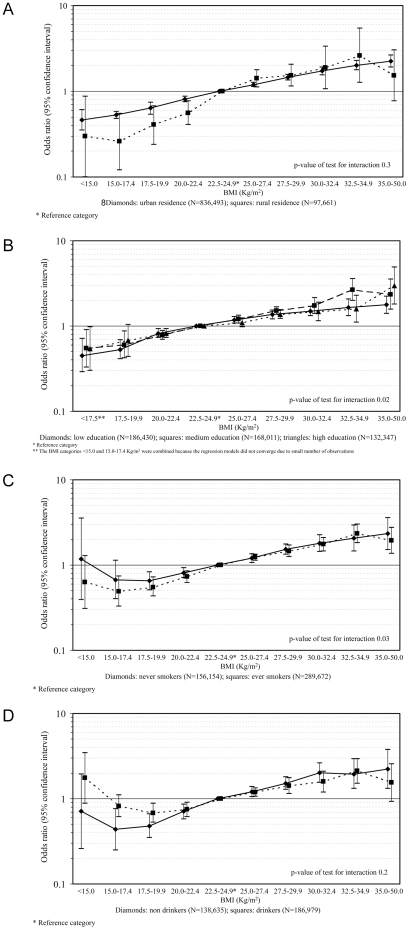
A. OR of diabetes for BMI category, stratified by urban/rural residence. B. OR of diabetes for BMI category, stratified by education. C. OR of diabetes for BMI category in men, stratified by tobacco smoking. D. OR of diabetes for BMI category in men, stratified by alcohol drinking

Figure 3 shows the results stratified by area of residence, education, tobacco smoking, and alcohol drinking. The association between BMI and prevalence of diabetes was suggested to be stronger in rural than in urban/suburban populations, though the rural population had a much lower diabetes prevalence in every BMI group (p-value for interaction 0.3; Figure 3A). The relationship was also stronger in subjects with less than the highest education than in subjects with high education (p-value for interaction 0.02; Figure 3B). Because the prevalence of tobacco smoking and alcohol drinking was low in women, the stratified analyses by these factors was restricted to men: the association between BMI and prevalence of diabetes was stronger in smokers than non-smokers (p-value 0.03; Figure 3C), no difference was observed between drinkers and non-drinkers (p-value 0.2; Figure 3D).

In all five cohorts with repeated BMI measurements during follow-up, individuals with diabetes experienced a decrease in BMI over time (cohort-specific mean change ranging from −0.06 to −0.2 kg/m^2^ yr), whereas in four of the five cohorts, the mean BMI increased among individuals without diabetes (range +0.007 to +0.3 kg/m^2^ yr; in the fifth cohort the mean BMI decreased by -0.02 kg/m^2^ yr). In all cohorts, the difference in BMI change between diabetic and non-diabetic individuals was statistically significant.

The summary results of the analysis based on ‘Asian’ cut-points are reported in Table S2. The results of the sensitivity analysis excluding the cohorts enrolled in the 1980s are reported in Table S3. They were similar to the results based on all cohorts.

## Discussion

This analysis of more than 900,000 adults from seven Asian countries confirmed the strong relationship between increased body mass and prevalence of diabetes; this has been observed in other populations, mainly in high-income countries, and has been interpreted as a causal association. Results were very similar no matter whether the standard or the ‘Asian’ categories of BMI were used. The association reported here for Asia, however, is somewhat weaker than that observed in high-income countries. A cohort study of nurses from the United States reported a 20-fold increased incidence of diabetes for obese women compared to those with BMI lower than 23 kg/m^2^ [20]. In our study, the ORs comparing obese and normal-weight individuals (i.e., BMI in the range 17.5–22.4 kg/m2) are in the order of 2.5–3: the difference with the nurses' study can hardly be explained by the use of prevalence data, or different BMI categories. Other studies from the US, however, reported more modest associations, with 6- to 8-fold increased incidence [21] or prevalence [22].

An elevated incidence [14] or prevalence [4], [12], [13], [23]–[27] of diabetes has been reported to be associated with high BMI in several previous studies from Asia. The difference in average BMI between diabetics and controls in these studies ranged between 2 and 4 kg/m^2^. In a multicentre study of 2,800 individuals from China, 6,300 from Japan and 3,300 from India, the age-adjusted prevalence of diabetes was 2-fold higher in China and 4-fold higher in Japan and India, across BMI categories 30+ vs. 15 kg/m^2^ [28]. In India, the association was stronger in women than in men. In two additional studies from Bangladesh (6,200 individuals) and Thailand (2,700 individuals), the adjusted prevalence of diabetes was compared across BMI categories. In the study from Bangladesh there was a 4-fold increased prevalence of diabetes (BMI 30+ vs. <16 kg/m^2^) in urban areas and a 6-fold increased prevalence in rural areas [29]; in the Thai study, a three-fold increased incidence was observed for BMI 28+ vs. <23 kg/m^2^ [30]. Our pooled analysis expands on these results and provides a systematic assessment of the association between body mass and diabetes prevalence in multiple populations across South and East Asia. Although our analysis included individuals with very low BMI (less than 17.5, and even less than 15 kg/m^2^), the number of cases of diabetes in these groups was relatively small, thus producing imprecise results.

The association between BMI and diabetes prevalence did not vary between men and women, but was stronger among younger than among older individuals. There are several possible explanations for this pattern, which has not been previously reported in large studies from Asia. First, genetic factors could play a more important role in those with earlier than later onset of diabetes, and the stronger association in younger adults could be due, in part, to joint effects of high BMI and such genetic factors. Second, the stronger association in younger adults may be due to a shorter latency of the effect of substantial weight gain, which is more likely to have occurred in those of younger age. A prospective cohort study in Japan also found that the effect of obesity on the risk of incident diabetes was greater for middle-aged than for older adults [31]. Analyses from a prospective study from Germany suggest that self-reported weight gain in early adulthood is related to a higher risk and an earlier onset of type 2 diabetes than is weight gain later [32]. Third, the interaction could be due to other factors that are associated with age, such as physical activity and dietary habits. Finally, it is possible that weight loss might be more substantial in older patients due to a relatively longer disease course and, as a result, the prevalent bias might be more substantial in older patients than younger patients, attenuating the positive association between BMI and diabetes prevalence. It should be noticed that this form of bias would affect more strongly the older age group, but would operate across the whole study population.

Although generally present in all subgroups of our study populations, the positive association between BMI and prevalence of diabetes differed in the countries where the cohorts were conducted. The association between low BMI and low diabetes prevalence was strongest in India and Bangladesh, intermediate in China, Taiwan, Korea, and Singapore, and weakest in Japan. This pattern was present in both sexes and in all age groups. Furthermore, the markedly lower diabetes prevalence among lean subjects was more evident in both Mumbai (an urban cohort) and Bangladesh (a rural cohort) than in other cohorts. In addition to a possible contribution of ethnic-specific genetic factors, it is plausible that low BMI may be a stronger indicator of low caloric diet throughout life – and, in particular, in early life – in lower- than in higher-income countries. It has been suggested that a high intake of white rice can disrupt glucose metabolism [33]-[34]. An explanation of the difference observed in the association between BMI and diabetes in Japan compared to the other countries may be a better clinical management of overweight and obese individuals: .because of wide dissemination of general health checkup in Japan in the past decades, it is possible that a sizable proportion of the Japanese subjects with history of diabetes have already reduced their weight at the time of entry into the cohort.

The pattern of results by tobacco smoking may be explained by an interaction between BMI and this habit in causing diabetes, possibly through the metabolic, inflammatory, and atherosclerotic consequences of tobacco [9]. In fact, current smokers had a lower BMI than former smokers and never smokers (median BMI levels were 22.3, 22.6 and 23.0 Kg/m^2^, respectively [p-value of difference<0.001]).

Our study provides further evidence that variation in the occurrence of diabetes in Asia and between Asia and other regions of the world cannot be fully explained by differences in body mass distribution among populations. Other anthropometric characteristics may play a role. Body fat distribution and, in particular, abdominal obesity, independent of body mass has been shown, in studies from Asia and other regions, to modify glucose metabolism and diabetes [27], [35]–[38]. Furthermore, Asian populations have been shown to exhibit a higher prevalence of abdominal obesity after adjusting for body mass [39]–[42]. Finally, there is evidence that waist circumference and other measures of abdominal obesity play a bigger role than BMI in determining diabetes risk in Asians as compared to populations of European descent [5], [43]–[44], but large-scale studies of unselected populations are needed to fully understand the shape of the dose-risk relation with different measures of adiposity. We have no information on this anthropometric characteristic.

It is plausible that other factors related to country of residence – either genetic or environmental (lifestyle factors and living conditions) – not only affect the overall risk of diabetes, but also modify the effect of established risk factors. There is some evidence that the role of reduced insulin secretion and reduced insulin sensitivity in determining diabetes risk may differ among populations. In particular, Indians seem to be more insulin-resistant than other populations [45]. Variation in intra-abdominal fat deposition and muscle mass could explain differences in the prevalence of diabetes and in the association between BMI and diabetes prevalence across Asian populations. An additional anthropometric characteristic that might modify the BMI-diabetes relation is low birth weight, an indicator of intrauterine (and probably infant) malnutrition. Low birth weight has been associated with glucose metabolism and risk of diabetes in studies in India [46]–[47], and the prevalence of low birth weight is higher in South Asia than in other regions of the continent [48]. The highest prevalence of diabetes in our study population was reported in Japan; there is some evidence that insulin deficiency rather than insulin resistance is an important factor in determining diabetes in Japanese individuals [49].

A limitation of our study is the use of self-reported diabetes status, which may have resulted in misclassification, leading to an underestimation of the association between BMI and diabetes prevalence, especially in low-income countries. However, available data on other indicators of diabetes in some of the cohorts supports the overall validity of these self-reported data. In this Asian population, glycosuria was measured in 11,122 (95%) members of the Bangladesh cohort, including 231 (95%) self-reported cases of diabetes: although diabetes treatment can influence this marker, more than 99% of non-cases tested negative for glycosuria and 62% of the cases tested positive. The result is comparable with that from high-income countries like Japan, where the sensitivity and specificity of the questionnaire for diabetic hyperglycaemia were 46% and 98%, respectively [14]. Furthermore, the age-adjusted prevalence in our study populations is consistent with results of other surveys conducted in most of the countries included in our study (see [5], [43] for reviews); one exception is the cohort from Mumbai, India, in which the prevalence of self-reported diabetes was lower than that reported from other surveys of urban populations from that country [5], [26], [50]–[51]. The use of self-reported information prevented us from distinguishing between type 1 and type 2 diabetes. Lack of information on BMI before diagnosis of diabetes is an additional limitation, since weight loss after the diagnosis (mainly for type 1, but also for type 2) would result in an underestimate of the role of BMI in determining the risk of the disease. However, prevalence of type 1 diabetes is substantially lower than type 2 diabetes in this region [3], and the inability to distinguish between types of diabetes almost certainly does not substantially affect the interpretation of the results. The reversed linear trend for the two lowest BMI groups (<15.0 and 15–17.4) in age<50 group is probably an effect of the presence of type 1 diabetes. Data from repeated measurements of BMI taken after the baseline in five of the cohorts show that BMI measured at baseline probably underestimated true BMI at the time of diagnosis of diabetes, thus reducing the measured strength of the association between body mass and prevalence of diabetes. The cross-sectional design of our study prevented us from studying the association between body mass and incidence of diabetes.

Our study provides a precise estimate of the shape and the strength of the association, in Asian populations, between body mass, as measured by BMI, and prevalence of diabetes, and identifies different patterns of risk depending on age, country, and other risk factors for diabetes.

## Supporting Information

Table S1 (DOCX)

Table S2 (DOCX)

Table S3 (DOCX)

References S1 (DOCX)
