# Supplementary material for: Body Mass Index and Diabetes in Asia: A Cross-Sectional Pooled Analysis of 900,000 Individuals in the Asia Cohort Consortium
Source: PLoS One. 2011 Jun 22;6(6):e19930. doi: 10.1371/journal.pone.0019930 (PMC3120751; doi:10.1371/journal.pone.0019930)
Supplement: Table S1 — (DOCX) [file pone.0019930.s001.docx]

Table S1: Cohort-specific odds ratios of diabetes for categories of body mass index, by sex

|  | | | | | | | | | | | |
| --- | --- | --- | --- | --- | --- | --- | --- | --- | --- | --- | --- |
| **Body mass index at baseline (Kg/m^2^)** | | | | | | | | | | | |
|  | **<15.0** | **15.0-17.4** | **17.5-19.9** | **20.0-22.4** | **22.5-24.9** | **25.0-27.4** | **27.5-29.9** | **30.0-32.4** | **32.5-34.9** | **35.0-50.0** | **Slope (SE)** |
| **Japan: 3 Prefecture Aichi Males (n=10,688)** | | | | | | | | | | | |
| N of cases (prevalence %) | 3 (12.0) | 20 (7.4) | 90 (5.2) | 198 (5.4) | 199 (6.1) | 87 (7.0) | 24 (8.0) | 13 (20.6) | 2 (14.3) | 1 (9.1) |  |
| OR | 1.33 | 0.88 | 0.73 | 0.85 | 1.00 | 1.22 | 1.30 | 4.41 | 2.63 | 1.30 | 0.066 |
| (95% CI) | (0.39,4.52) | (0.54,1.43) | (0.56,0.94) | (0.70,1.05) | (reference) | (0.94,1.59) | (0.83,2.02) | (2.33,8.34) | (0.57,12.0) | (0.16,10.4) | (0.014) |
|  |  |  |  |  |  |  |  |  |  |  |  |
| **Japan: 3 Prefecture Aichi Females (n=11,953)** | | | | | | | | | | | |
| N of cases (prevalence %) | 3 (7.5) | 14 (2.8) | 59 (2.2) | 127 (3.0) | 107 (3.6) | 58 (5.4) | 24 (7.4) | 10 (10.6) | 2 (10.5) | 1 (12.5) |  |
| OR | 1.08 | 0.49 | 0.53 | 0.88 | 1.00 | 1.39 | 1.89 | 2.89 | 2.68 | 2.29 | 0.111 |
| (95% CI) | (0.32,3.59) | (0.28,0.88) | (0.38,0.74) | (0.78,1.15) | (reference) | (1.00,1.93) | (1.19,3.02) | (1.44,5.80) | (0.59,12.2) | (0.37,19.5) | (0.015) |
|  |  |  |  |  |  |  |  |  |  |  |  |
| **Japan: Ibaraki Males (n=33,336)** | | | | | | | | | | | |
| No of cases of diabetes | 0 (0.0) | 23 (4.3) | 115 (3.0) | 332 (3.7) | 431 (4.0) | 282 (4.4) | 99 (4.6) | 23 (5.0) | 8 (9.5) | 1 (4.4) |  |
|  |  | 0.89 | 0.65 | 0.87 | 1.00 | 1.12 | 1.20 | 1.41 | 2.86 | 1.27 | 0.059 |
| OR (95% CI) |  | (0.58,1.37) | (0.53,0.80) | (0.75,1.00) | (reference) | (0.96,1.31) | (0.96,1.50) | (0.92,2.18) | (1.36,6.01) | (0.17,9.56) | (0.009) |
|  |  |  |  |  |  |  |  |  |  |  |  |
| **Japan: Ibaraki Females (n=64,272)** | | | | | | | | | | | |
| N of cases (prevalence %) | 1 (2.3) | 18 (1.8) | 107 (1.6) | 290 (1.7) | 441 (2.2) | 344 (2.8) | 164 (3.2) | 56 (3.6) | 22 (5.1) | 12 (7.1) |  |
| OR | 0.73 | 0.68 | 0.72 | 0.81 | 1.00 | 1.16 | 1.29 | 1.53 | 2.35 | 3.71 | 0.071 |
| (95% CI) | (0.10,5.32) | (0.42,1.09) | (0.58,0.89) | (0.70,0.94) | (reference) | (1.01,1.34) | (1.07,1.54) | (1.15,2.03) | (1.51,3.66) | (2.03,6.77) | (0.008) |
|  |  |  |  |  |  |  |  |  |  |  |  |
| **Japan: JACC Males (n=31,697)** | | | | | | | | | | | |
| N of cases (prevalence %) | 3 (9.1) | 50 (8.6) | 309 (6.8) | 707 (6.5) | 654 (6.6) | 345 (8.0) | 104 (8.9) | 19 (7.5) | 3 (5.8) | 1 (4.2) |  |
| OR | 1.01 | 0.99 | 0.91 | 0.95 | 1.00 | 1.28 | 1.49 | 1.24 | 0.89 | 0.58 | 0.031 |
| (95% CI) | (0.30,3.32) | (0.73,1.35) | (0.79,1.05) | (0.85,1.06) | (reference) | (1.12,1.47) | (1.20,1.85) | (0.77,1.99) | (0.28,2.89) | (0.08,4.34) | (0.007) |
|  |  |  |  |  |  |  |  |  |  |  |  |
| **Japan: JACC Females (n=43,789)** | | | | | | | | | | | |
| N of cases (prevalence %) | 2 (2.5) | 46 (4.5) | 229 (3.6) | 524 (4.0) | 568 (4.2) | 321 (4.8) | 144 (6.3) | 62 (9.3) | 13 (8.1) | 9 (9.1) |  |
| OR | 0.33 | 0.73 | 0.73 | 0.93 | 1.00 | 1.10 | 1.48 | 2.21 | 2.00 | 2.12 | 0.039 |
| (95% CI) | (0.08,1.35) | (0.54,1.00) | (0.63,0.86) | (0.82,1.05) | (reference) | (0.95,1.26) | (1.22,1.79) | (1.67,2.92) | (1.12,3.58) | (1.05,4.27) | (0.007) |
|  |  |  |  |  |  |  |  |  |  |  |  |
| **Japan: JPHC1 Males (n=20,464)** | | | | | | | | | | | |
| N of cases (prevalence %) | 0 (0.0) | 6 (5.8) | 106 (6.2) | 274 (4.7) | 389 (5.4) | 248 (6.2) | 84 (6.7) | 22 (7.0) | 6 (7.1) | 4 (18.2) |  |
| OR |  | 1.07 | 1.15 | 0.86 | 1.00 | 1.16 | 1.26 | 1.32 | 1.34 | 3.40 | 0.034 |
| (95% CI) |  | (0.47,2.47) | (0.92,1.43) | (0.73,1.01) | (reference) | (0.99,1.37) | (0.98,1.60) | (0.84,2.06) | (0.58,3.09) | (1.14,10.1) | (0.011) |
|  |  |  |  |  |  |  |  |  |  |  |  |
| **Japan: JPHC1 Females (n=22,307)** | | | | | | | | | | | |
| N of cases (prevalence %) | 0 (0.0) | 5 (2.2) | 35 (1.6) | 126 (2.1) | 155 (2.1) | 146 (3.5) | 61 (3.7) | 23 (4.4) | 2 (1.5) | 6 (10.2) |  |
| OR |  | 1.01 | 0.76 | 1.02 | 1.00 | 1.61 | 1.71 | 2.02 | 0.67 | 4.78 | 0.073 |
| (95% CI) |  | (0.41,2.50) | (0.52,1.10) | (0.80,1.29) | (reference) | (1.28,2.03) | (1.26,2.31) | (1.29,3.16) | (0.16,2.72) | (2.01,11.3) | (0.013) |
|  |  |  |  |  |  |  |  |  |  |  |  |
| **Japan: JPHC2 Males (n=26,419)** | | | | | | | | | | | |
| N of cases (prevalence %) | 1 (5.9) | 22 (8.5) | 178 (7.1) | 531 (7.2) | 719 (8.0) | 391 (7.9) | 138 (8.2) | 48 (10.5) | 12 (14.6) | 2 (6.1) |  |
| OR | 0.57 | 0.91 | 0.85 | 0.88 | 1.00 | 1.02 | 1.08 | 1.46 | 2.12 | 0.82 | 0.032 |
| (95% CI) | (0.08,4.34) | (0.58,1.42) | (0.72,1.01) | (0.78,0.99) | (reference) | (0.90,1.16) | (0.89,1.31) | (1.07,2.00) | (1.14,3.94) | (0.20,3.47) | (0.008) |
|  |  |  |  |  |  |  |  |  |  |  |  |
| **Japan: JPHC2 Females (n=29,293)** | | | | | | | | | | | |
| N of cases (prevalence %) | 1 (3.8) | 15 (3.6) | 106 (3.2) | 236 (2.9) | 298 (3.3) | 236 (4.6) | 121 (6.0) | 58 (8.6) | 17 (8.4) | 10 (11.8) |  |
| OR | 1.01 | 1.00 | 0.99 | 0.92 | 1.00 | 1.35 | 1.77 | 2.67 | 2.70 | 3.84 | 0.082 |
| (95% CI) | (0.14,7.55) | (0.59,1.70) | (0.79,1.25) | (0.77,1.10) | (reference) | (1.14,1.61) | (1.42,2.20) | (1.99,3.58) | (1.62,4.51) | (1.95,7.53) | (0.009) |
|  |  |  |  |  |  |  |  |  |  |  |  |
| **Japan: 3 Prefecture Miyagi Males (n=13,297)** | | | | | | | | | | | |
| N of cases (prevalence %) | 3 (21.4) | 14 (6.1) | 111 (7.1) | 284 (7.0) | 342 (7.7) | 201 (9.5) | 54 (8.6) | 16 (10.7) | 1 (3.7) | 6 (10.0) |  |
| OR | 2.70 | 0.68 | 0.85 | 0.88 | 1.00 | 1.26 | 1.14 | 1.46 | 0.45 | 1.23 | 0.035 |
| (95% CI) | (0.75,9.75) | (0.39,1.19) | (0.68,1.07) | (0.74,1.03) | (reference) | (1.05,1.52) | (0.84,1.54) | (0.86,2.48) | (0.06,3.32) | (0.53,2.90) | (0.009) |
|  |  |  |  |  |  |  |  |  |  |  |  |
| **Japan: 3 Prefecture Miyagi Females (n=16,257)** | | | | | | | | | | | |
| N of cases (prevalence %) | 0 (0.0) | 14 (3.8) | 75 (3.4) | 132 (3.0) | 195 (4.2) | 133 (4.7) | 70 (5.9) | 30 (8.0) | 8 (7.6) | 8 (8.3) |  |
| OR |  | 0.64 | 0.71 | 0.72 | 1.00 | 1.10 | 1.31 | 1.76 | 1.64 | 1.69 | 0.054 |
| (95% CI) |  | (0.37,1.12) | (0.54,0.94) | (0.58,0.91) | (reference) | (0.87,1.38) | (0.99,1.74) | (1.17,2.63) | (0.78,3.45) | (0.80,3.56) | (0.009) |
|  |  |  |  |  |  |  |  |  |  |  |  |
| **Japan: Miyagi Males (n=21,504)** | | | | | | | | | | | |
| N of cases (prevalence %) | 1 (12.5) | 10 (8.0) | 96 (5.5) | 313 (5.0) | 380 (5.0) | 237 (5.9) | 93 (6.8) | 33 (9.3) | 4 (7.4) | 2 (7.1) |  |
| OR | 2.31 | 1.49 | 1.07 | 1.00 | 1.00 | 1.29 | 1.44 | 2.02 | 1.61 | 1.41 | 0.037 |
| (95% CI) | (0.28,19.1) | (0.77,2.88) | (0.85,1.34) | (0.86,1.17) | (reference) | (1.01,1.42) | (1.14,1.83) | (1.39,2.94) | (0.57,4.50) | (0.33,6.02) | (0.010) |
|  |  |  |  |  |  |  |  |  |  |  |  |
| **Japan: Miyagi Females (n=23,364)** | | | | | | | | | | | |
| N of cases (prevalence %) | 0 (0.0) | 4 (1.9) | 48 (2.2) | 175 (2.8) | 226 (3.0) | 157 (3.4) | 93 (4.8) | 30 (5.4) | 8 (6.8) | 5 (6.7) |  |
| OR |  | 0.56 | 0.76 | 1.00 | 1.00 | 1.07 | 1.52 | 1.74 | 2.14 | 2.08 | 0.061 |
| (95% CI) |  | (0.21,1.52) | (0.55,1.04) | (0.82,1.22) | (reference) | (0.87,1.32) | (1.18,1.94) | (1.17,2.57) | (1.03,4.46) | (0.83,5.23) | (0.011) |
|  |  |  |  |  |  |  |  |  |  |  |  |
| **Japan: Ohsaki Males (n=23,000)** | | | | | | | | | | | |
| N of cases (prevalence %) | 1 (5.0) | 23 (7.2) | 155 (6.5) | 451 (6.6) | 613 (8.0) | 327 (8.2) | 128 (9.7) | 36 (10.6) | 9 (12.5) | 5 (10.6) |  |
| OR | 0.48 | 0.76 | 0.75 | 0.80 | 1.00 | 1.07 | 1.33 | 1.48 | 1.88 | 1.33 | 0.042 |
| (95% CI) | (0.06,3.64) | (0.49,1.17) | (0.62,0.90) | (0.71,0.91) | (reference) | (0.93,1.23) | (1.09,1.63) | (1.03,2.12) | (0.93,3.82) | (0.52,3.38) | (0.007) |
|  |  |  |  |  |  |  |  |  |  |  |  |
| **Japan: Ohsaki Females (n=24,710)** | | | | | | | | | | | |
| N of cases (prevalence %) | 3 (5.7) | 14 (3.5) | 121 (4.8) | 329 (5.3) | 418 (5.4) | 306 (6.3) | 142 (6.8) | 56 (8.9) | 18 (10.8) | 15 (13.3) |  |
| OR | 0.67 | 0.49 | 0.80 | 0.98 | 1.00 | 1.12 | 1.24 | 1.63 | 2.04 | 2.38 | 0.045 |
| (95% CI) | (0.21,2.18) | (0.28,0.84) | (0.65,0.99) | (0.85,1.14) | (reference) | (0.96,1.31) | (1.02,1.51) | (1.21,2.18) | (1.23,3.37) | (1.36,4.17) | (0.007) |
|  |  |  |  |  |  |  |  |  |  |  |  |
| **China: CHEFS Males (n=70,854)** | | | | | | | | | | | |
| N of cases (prevalence %) | 1 (0.4) | 17 (0.5) | 83 (0.6) | 190 (0.9) | 422 (2.6) | 404 (4.2) | 237 (6.4) | 81 (7.4) | 13 (5.8) | 6 (4.3) |  |
| OR | 0.14 | 0.18 | 0.21 | 0.34 | 1.00 | 1.65 | 2.47 | 2.84 | 2.19 | 1.59 | 0.176 |
| (95% CI) | (0.02,0.99) | (0.11,0.30) | (0.17,0.27) | (0.29,0.41) | (reference) | (1.44,1.90) | (2.10,2.91) | (2.23,3.65) | (1.24,3.86) | (0.70,3.63) | (0.006) |
|  |  |  |  |  |  |  |  |  |  |  |  |
| **China: CHEFS Females (n=73,030)** | | | | | | | | | | | |
| N of cases (prevalence %) | 4 (0.7) | 20 (0.5) | 85 (0.6) | 222 (1.2) | 351 (2.1) | 400 (3.9) | 258 (4.9) | 140 (6.5) | 51 (8.0) | 40 (8.8) |  |
| OR | 0.34 | 0.22 | 0.29 | 0.58 | 1.00 | 1.84 | 2.31 | 3.01 | 3.73 | 4.20 | 0.134 |
| (95% CI) | (0.13,0.92) | (0.14,0.34) | (0.23,0.36) | (0.49,0.69) | (reference) | (1.60,2.14) | (1.96,2.72) | (2.46,3.68) | (2.75,5.06) | (2.98,5.92) | (0.005) |
|  |  |  |  |  |  |  |  |  |  |  |  |
| **China: SCS Males (n=18,100)** | | | | | | | | | | | |
| N of cases (prevalence %) | 1 (3.9) | 8 (1.2) | 20 (0.5) | 51 (0.9) | 83 (1.8) | 40 (1.8) | 20 (3.1) | 7 (4.7) | 2 (11.8) | 1 (12.5) |  |
| OR | 2.04 | 0.64 | 0.28 | 0.46 | 1.00 | 0.94 | 1.59 | 2.44 | 6.41 | 7.05 | 0.154 |
| (95% CI) | (0.27,15.3) | (0.31,1.32) | (0.17,0.46) | (0.35,0.66) | (reference) | (0.64,1.38) | (0.97,2.62) | (1.10,5.37) | (1.44,28.6) | (0.85,58.7) | (0.020) |
|  |  |  |  |  |  |  |  |  |  |  |  |
| **China: SMHS Males (n=61,379)** | | | | | | | | | | | |
| N of cases (prevalence %) | 1 (2.6) | 19 (1.9) | 217 (3.6) | 795 (5.6) | 1316 (6.6) | 933 (6.8) | 427 (8.7) | 107 (8.5) | 23 (9.5) | 8 (12.9) |  |
| OR | 0.25 | 0.26 | 0.57 | 0.86 | 1.00 | 0.99 | 1.24 | 1.18 | 1.33 | 1.98 | 0.058 |
| (95% CI) | (0.03,1.86) | (0.17,0.42) | (0.49,0.66) | (0.79,0.95) | (reference) | (0.91,1.08) | (1.11,1.40) | (0.96,1.45) | (0.86,2.07) | (0.93,4.23) | (0.005) |
|  |  |  |  |  |  |  |  |  |  |  |  |
| **China: SWHS Females (n=74,881)** | | | | | | | | | | | |
| N of cases (prevalence %) | 1 (2.2) | 25 (2.7) | 176 (2.5) | 519 (2.8) | 888 (4.0) | 843 (5.5) | 498 (6.8) | 233 (8.7) | 70 (9.0) | 45 (11.7) |  |
| OR | 0.34 | 0.59 | 0.74 | 0.82 | 1.00 | 1.18 | 1.32 | 1.60 | 1.60 | 2.12 | 0.060 |
| (95% CI) | (0.05,2.47) | (0.39,0.88) | (0.63,0.87) | (0.74,0.92) | (reference) | (1.07,1.31) | (1.18,1.48) | (1.37,1.86) | (1.23,2.07) | (1.53,2.93) | (0.005) |
|  |  |  |  |  |  |  |  |  |  |  |  |
| **Taiwan: CBCSP Males (n=11,934)** | | | | | | | | | | | |
| N of cases (prevalence %) | 0 (0.0) | 2 (1.8) | 16 (1.5) | 61 (2.1) | 93 (2.6) | 93 (3.5) | 46 (4.2) | 16 (4.7) | 5 (6.3) | 1 (3.6) |  |
| OR |  | 0.64 | 0.59 | 0.80 | 1.00 | 1.30 | 1.54 | 1.80 | 2.72 | 1.70 | 0.087 |
| (95% CI) |  | (0.15,2.65) | (0.34,1.00) | (0.58,1.11) | (reference) | (0.97,1.75) | (1.07,2.21) | (1.04,3.11) | (1.06,7.00) | (0.22,12.9) | (0.017) |
|  |  |  |  |  |  |  |  |  |  |  |  |
| **Taiwan: CBCSP Females (n=11,769)** | | | | | | | | | | | |
| N of cases (prevalence %) | 0 (0.0) | 1 (0.7) | 9 (0.8) | 37 (1.3) | 76 (2.3) | 67 (2.8) | 47 (3.8) | 14 (3.0) | 8 (5.4) | 3 (3.7) |  |
| OR |  | 0.37 | 0.49 | 0.71 | 1.00 | 1.11 | 1.45 | 1.07 | 2.07 | 1.52 | 0.076 |
| (95% CI) |  | (0.05,2.74) | (0.24,0.98) | (0.47,1.05) | (reference) | (0.80,1.56) | (1.00,2.11) | (0.60,1.92) | (0.97,4.42) | (0.46,4.99) | (0.016) |
|  |  |  |  |  |  |  |  |  |  |  |  |
| **Taiwan: CVDFACTS Males (n=2262)** | | | | | | | | | | | |
| N of cases (prevalence %) | 0 (0.0) | 0 (0.0) | 1 (0.4) | 14 (2.4) | 21 (3.1) | 19 (4.0) | 12 (6.6) | 4 (7.1) | 1 (5.3) | 1 (12.5) |  |
| OR |  |  | 0.17 | 0.95 | 1.00 | 1.26 | 2.11 | 2.56 | 2.46 | 5.82 | 0.130 |
| (95% CI) |  |  | (0.02,1.29) | (0.48,1.91) | (reference) | (0.66,2.39) | (1.00,4.44) | (0.82,7.96) | (0.28,21.2) | (0.60,56.8) | (0.036) |
|  |  |  |  |  |  |  |  |  |  |  |  |
| **Taiwan: CVDFACTS Females (n=2867)** | | | | | | | | | | | |
| N of cases (prevalence %) | 0 (0.0) | 1 (1.8) | 5 (1.3) | 13 (1.8) | 31 (3.9) | 24 (4.8) | 21 (7.9) | 5 (5.0) | 2 (5.6) | 2 (11.8) |  |
| OR |  | 0.98 | 0.78 | 0.64 | 1.00 | 0.95 | 1.70 | 1.03 | 1.08 | 2.69 | 0.078 |
| (95% CI) |  | (0.12,7.85) | (0.29,2.08) | (0.33,1.25) | (reference) | (0.54,1.65) | (0.94,3.05) | (0.38,2.75) | (0.24,4.82) | (0.55,13.1) | (0.028) |
|  |  |  |  |  |  |  |  |  |  |  |  |
| **Korea: KMCC Males (n=5988)** | | | | | | | | | | | |
| N of cases (prevalence %) | 2 (20.0) | 5 (4.5) | 28 (3.6) | 59 (3.3) | 107 (5.8) | 61 (5.9) | 23 (6.9) | 3 (3.8) | 0 (0.0) | 1 (25.0) |  |
| OR | 3.02 | 0.64 | 0.53 | 0.54 | 1.00 | 1.07 | 1.28 | 0.75 |  | 5.63 | 0.088 |
| (95% CI) | (0.62,14.6) | (0.25,1.60) | (0.34,0.81) | (0.39,0.74) | (reference) | (0.77,1.48) | (0.80,2.05) | (0.23,2.45) |  | (0.56,56.8) | (0.020) |
|  |  |  |  |  |  |  |  |  |  |  |  |
| **Korea: KMCC Females (n=9070)** | | | | | | | | | | | |
| N of cases (prevalence %) | 0 (0.0) | 3 (2.2) | 23 (2.7) | 64 (3.1) | 138 (5.0) | 113 (5.7) | 73 (8.5) | 18 (5.6) | 9 (11.5) | 2 (7.4) |  |
| OR |  | 0.34 | 0.46 | 0.56 | 1.00 | 1.14 | 1.70 | 1.17 | 3.01 | 1.88 | 0.105 |
| (95% CI) |  | (0.11,1.09) | (0.29,0.72) | (0.41,0.76) | (reference) | (0.88,1.47) | (1.26,2.29) | (0.70,1.96) | (1.45,6.24) | (0.43,8.18) | (0.014) |
|  |  |  |  |  |  |  |  |  |  |  |  |
| **Singapore: SCHS Males (n=27,954)** | | | | | | | | | | | |
| N of cases (prevalence %) | 3 (3.8) | 23 (2.8) | 182 (5.1) | 498 (7.1) | 933 (9.0) | 459 (11.9) | 215 (14.5) | 74 (14.8) | 24 (20.3) | 14 (18.4) |  |
| OR | 0.32 | 0.27 | 0.53 | 0.78 | 1.00 | 1.47 | 1.89 | 1.97 | 2.88 | 2.51 | 0.109 |
| (95% CI) | (0.10,1.03) | (0.18,0.41) | (0.45,0.63) | (0.70,0.88) | (reference) | (1.30,1.65) | (1.61,2.22) | (1.42,2.55) | (1.82,4.56) | (1.39,4.53) | (0.006) |
|  |  |  |  |  |  |  |  |  |  |  |  |
| **Singapore: SCHS Females (n=35,303)** | | | | | | | | | | | |
| N of cases (prevalence %) | 7 (7.2) | 41 (4.1) | 240 (5.5) | 605 (7.5) | 1449 (10.3) | 492 (11.0) | 261 (12.8) | 116 (13.5) | 38 (16.7) | 22 (13.7) |  |
| OR | 0.60 | 0.37 | 0.57 | 0.80 | 1.00 | 1.16 | 1.35 | 1.43 | 1.85 | 1.48 | 0.070 |
| (95% CI) | (0.28,1.30) | (0.27,0.51) | (0.49,0.66) | (0.73,0.89) | (reference) | (1.04,1.30) | (1.17,1.55) | (1.16,1.75) | (1.29,2.65) | (0.94,2.36) | (0.005) |
|  |  |  |  |  |  |  |  |  |  |  |  |
| **Bangladesh: HEALS Males (n=4744)** | | | | | | | | | | | |
| N of cases (prevalence %) | 2 (1.8) | 10 (0.9) | 23 (1.2) | 25 (2.7) | 29 (6.9) | 21 (10.7) | 6 (9.8) | 1 (6.2) | 1 (33.3) | 0 (0.0) |  |
| OR | 0.17 | 0.11 | 0.18 | 0.39 | 1.00 | 1.84 | 1.45 | 0.94 | 8.42 |  | 0.229 |
| (95% CI) | (0.04,0.75) | (0.05,0.23) | (0.10,0.31) | (0.22,0.67) | (reference) | (1.01,3.35) | (0.57,3.70) | (0.12,7.50) | (0.71,100) |  | (0.024) |
|  |  |  |  |  |  |  |  |  |  |  |  |
| **Bangladesh: HEALS Females (n=6418)** | | | | | | | | | | | |
| N of cases (prevalence %) | 2 (1.2) | 8 (0.6) | 23 (1.0) | 25 (1.7) | 31 (4.1) | 20 (6.1) | 3 (2.4) | 5 (13.9) | 1 (6.7) | 0 (0.0) |  |
| OR | 0.22 | 0.14 | 0.24 | 0.40 | 1.00 | 1.51 | 0.52 | 3.09 | 1.57 |  | 0.177 |
| (95% CI) | (0.05,0.93) | (0.06,0.30) | (0.14,0.42) | (0.23,0.68) | (reference) | (0.84,2.69) | (0.16,1.74) | (1.08,8.83) | (0.20,12.5) |  | (0.021) |
|  |  |  |  |  |  |  |  |  |  |  |  |
| **India: MSC Males (n=69,165)** | | | | | | | | | | | |
| N of cases (prevalence %) | 12 (0.9) | 37 (0.6) | 206 (1.5) | 523 (2.8) | 526 (3.4) | 371 (4.2) | 186 (5.5) | 74 (5.6) | 28 (7.0) | 13 (7.9) |  |
| OR | 0.21 | 0.15 | 0.42 | 0.81 | 1.00 | 1.29 | 1.65 | 1.75 | 2.17 | 2.24 | 0.123 |
| (95% CI) | (0.12,0.37) | (0.11,0.22) | (0.36,0.49) | (0.72,0.92) | (reference) | (1.13,1.48) | (1.39,1.96) | (1.36,2.26) | (1.45,3.23) | (1.25,3.99) | (0.006) |
|  |  |  |  |  |  |  |  |  |  |  |  |
| **India: MCS Females (n=32,100)** | | | | | | | | | | | |
| N of cases (prevalence %) | 1 (0.1) | 12 (0.4) | 32 (0.6) | 65 (1.0) | 114 (1.8) | 100 (2.1) | 58 (2.3) | 31 (2.2) | 16 (3.4) | 7 (2.2) |  |
| OR | 0.05 | 0.20 | 0.32 | 0.57 | 1.00 | 1.22 | 1.24 | 1.23 | 1.93 | 1.16 | 0.103 |
| (95% CI) | (0.01,0.34) | (0.11,0.37) | (0.21,0.47) | (0.42,0.77) | (reference) | (0.93,1.60) | (0.90,1.71) | (0.82,1.84) | (1.13,3.30) | (0.53,2.52) | (0.009) |
|  |  |  |  |  |  |  |  |  |  |  |  |

OR, odds ratio, adjusted for age; CI, confidence interval; SE, standard error
