# Supplementary material for: Body Mass Index and Diabetes in Asia: A Cross-Sectional Pooled Analysis of 900,000 Individuals in the Asia Cohort Consortium
Source: PLoS One. 2011 Jun 22;6(6):e19930. doi: 10.1371/journal.pone.0019930 (PMC3120751; doi:10.1371/journal.pone.0019930)
Supplement: Table S2 — (DOCX) [file pone.0019930.s002.docx]

Table S2. Odds ratios of diabetes for body mass index, overall and stratified by sex and age - 'Asian' BMI cutpoints.

|  | | | | | | |
| --- | --- | --- | --- | --- | --- | --- |
| **Body mass index at baseline (Kg/m^2^)** | | | | | | |
|  | **<18.5** | **18.5-22.9** | **23.0-24.9** | **25.0-29.9** | **30.0-50.0** | **Slope (SE)** |
| **All subjects (n =934,154)^a^** | | | | | | |
| N of cases (prevalence %) | 1407 (2.0) | 13857 (3.4) | 10085 (4.7) | 12300 (5.6) | 2145 (7.5) |  |
| OR ^a^ | 0.51 | 0.71 | 1.00 | 1.25 | 1.79 | 0.084 |
| (95% CI) | (0.37,0.69) | (0.60,0.84) | (reference) | (1.17,1.33) | (1.59,2.01) | (0.010) |
|  | | | | | | |
| **Men (n=452,785)** | | | | | | |
| N of cases (prevalence %) | 812 (2.3) | 8319 (4.1) | 5639 (5.4) | 6467 (6.4) | 786 (8.3) |  |
| OR ^a^ | 0.54 | 0.71 | 1.00 | 1.25 | 1.73 | 0.084 |
| (95% CI) | (0.37,0.79) | (0.58,0.86) | (reference) | (1.15,1.36) | (1.48,2.01) | (0.013) |
|  | | | | | | |
| **Women (n=481,369)** | | | | | | |
| N of cases (prevalence %) | 595 (1.7) | 5538 (2.8) | 4446 (4.1) | 5833 (4.9) | 1359 (7.2) |  |
| OR ^a^ | 0.48 | 0.73 | 1.00 | 1.28 | 1.84 | 0.083 |
| (95% CI) | (0.35,0.67) | (0.62,0.85) | (reference) | (1.17,1.40) | (1.57,2.16) | (0.008) |
|  | | | | | | |
| **Age<50 (n=334,515)** | | | | | | |
| N of cases (prevalence %) | 167 (0.7) | 2002 (1.3) | 1500 (2.0) | 2094 (2.9) | 419 (4.7) |  |
| OR ^a^ | 0.54 | 0.67 | 1.00 | 1.46 | 2.71 | 0.117* |
| (95% CI) | (0.36,0.81) | (0.56,0.81) | (reference) | (1.30,1.63) | (2.20,3.33) | (0.010) |
|  | | | | | | |
| **Age 50-59 (n=292,225)** | | | | | | |
| N of cases (prevalence %) | 376 (2.1) | 4278 (3.5) | 3308 (4.7) | 4085 (5.6) | 739 (7.7) |  |
| OR ^a^ | 0.59 | 0.75 | 1.00 | 1.25 | 1.87 | 0.082* |
| (95% CI) | (0.39,0.88) | (0.63,0.89) | (reference) | (1.16,1.35) | (1.62,2.16) | (0.013) |
|  | | | | | | |
| **Age≥60 (n=307,414)** | | | | | | |
| N of cases (prevalence %) | 864 (3.1) | 7577 (5.9) | 5277 (7.8) | 6121 (8.4) | 987 (9.9) |  |
| OR ^a^ | 0.48 | 0.76 | 1.00 | 1.19 | 1.47 | 0.076* |
| (95% CI) | (0.36,0.64) | (0.66.0.87) | (reference) | (1.12,1.26) | (1.32,1.63) | (0.011) |
|  |  |  |  |  |  |  |

^a^ Meta-analysis estimates of cohort-specific OR adjusted for age and sex.

* P-value of difference across strata <0.001

OR, odds ratio; CI, confidence interval; SE, standard error
