# Supplementary material for: Body Mass Index and Diabetes in Asia: A Cross-Sectional Pooled Analysis of 900,000 Individuals in the Asia Cohort Consortium
Source: PLoS One. 2011 Jun 22;6(6):e19930. doi: 10.1371/journal.pone.0019930 (PMC3120751; doi:10.1371/journal.pone.0019930)
Supplement: Table S3 — (DOCX) [file pone.0019930.s004.docx]

Table S3: Odds ratios of diabetes for body mass index, overall and stratified by sex and age. Without 3 cohorts which were established in the 1980’s.

|  | | | | | | | | | | | |
| --- | --- | --- | --- | --- | --- | --- | --- | --- | --- | --- | --- |
| **Body mass index at baseline (Kg/m^2^)** | | | | | | | | | | | |
|  | **<15.0** | **15.0-17.4** | **17.5-19.9** | **20.0-22.4** | **22.5-24.9** | **25.0-27.4** | **27.5-29.9** | **30.0-32.4** | **32.5-34.9** | **35.0-50.0** | **Slope (SE)** |
|  | | | | | | | | | | | |
| **All subjects (n =863,859)^a^** | | | | | | | | | | | |
| N of cases (prevalence %) | 49 (1.2) | 460 (1.6) | 2954 (2.5) | 8003 (3.5) | 11817 (4.7) | 7760 (5.3) | 3642 (6.4) | 1388 (7.1) | 412 (8.0) | 237 (8.7) |  |
| OR ^a^ | 0.39 | 0.46 | 0.58 | 0.76 | 1.00 | 1.22 | 1.47 | 1.67 | 2.05 | 2.29 | 0.083 |
| (95% CI) | (0.25,0.62) | (0.31,0.69) | (0.45,0.75) | (0.66,0.88) | (reference) | (1.12,1.32) | (1.32,1.63) | (1.45,1.92) | (1.73,2.42) | (1.89,2.77) | (0.011) |
|  |  |  |  |  |  |  |  |  |  |  |  |
| **Men (n=410,700)** | | | | | | | | | | | |
| N of cases (prevalence %) | 27 (1.4) | 247 (1.7) | 1715 (2.9) | 4773 (4.2) | 6633 (5.5) | 4191 (6.2) | 1798 (7.4) | 541 (7.9) | 137 (9.0) | 59 (8.9) |  |
| OR ^a^ | 0.45 | 0.48 | 0.57 | 0.75 | 1.00 | 1.22 | 1.48 | 1.63 | 2.02 | 1.99 | 0.084 |
| (95% CI) | (0.23,0.87) | (0.30,0.78) | (0.43,0.77) | (0.45,0.89) | (reference) | (1.11,1.33) | (1.29,1.69) | (1.38,1.93) | (1.65,2.47) | (1.51,2.63) | (0.015) |
|  |  |  |  |  |  |  |  |  |  |  |  |
| **Women (n=453,159)** | | | | | | | | | | | |
| N of cases (prevalence %)s | 22 (1.1) | 213 (1.5) | 1239 (2.1) | 3230 (2.8) | 5184 (3.9) | 3569 (4.6) | 1944 (5.6) | 847 (6.7) | 275 (7.6) | 178 (8.7) |  |
| OR ^a^ | 0.42 | 0.46 | 0.58 | 0.78 | 1.00 | 1.24 | 1.49 | 1.74 | 2.16 | 2.48 | 0.081 |
| (95% CI) | (0.27,0.65) | (0.33,0.63) | (0.47,0.72) | (0.69,0.88) | (reference) | (1.13,1.36) | (1.34,1.67) | (1.47,2.07) | (1.90,2.46) | (1.90,3.22) | (0.009) |
|  |  |  |  |  |  |  |  |  |  |  |  |
| **Age<50 (n=313,146)** | | | | | | | | | | | |
| N of cases (prevalence %) | 6 (0.5) | 52 (0.5) | 420 (0.9) | 1154 (1.3) | 1708 (1.9) | 1267 (2.6) | 662 (3.5) | 282 (4.6) | 81 (4.9) | 38 (4.6) |  |
| OR ^a^ | 2.16 | 0.63 | 0.58 | 0.69 | 1.00 | 1.35 | 1.92 | 2.65 | 3.35 | 3.99 | 0.117* |
| (95% CI) | (0.55,8.45) | (0.38,1.03) | (0.45,0.75) | (0.59,0.81) | (reference) | (1.18,1.53) | (1.62,2.30) | (2.15,3.27) | (2.49,4.51) | (2.85,5.58) | (0.011) |
|  |  |  |  |  |  |  |  |  |  |  |  |
| **Age 50-59 (n=265,865)** | | | | | | | | | | | |
| N of cases (prevalence %) | 10 (1.2) | 111 (1.6) | 874 (2.7) | 2465 (3.6) | 3829 (4.7) | 2597 (5.4) | 1208 (6.1) | 472 (7.2) | 144 (8.5) | 85 (9.7) |  |
| OR ^a^ | 0.65 | 0.61 | 0.65 | 0.77 | 1.00 | 1.21 | 1.41 | 1.69 | 2.26 | 2.79 | 0.080* |
| (95% CI) | (0.23,1.80) | (0.33,1.12) | (0.49,0.86) | (0.65,0.91) | (reference) | (1.11,1.32) | (1.25,1.60) | (1.44,1.99) | (1.84,2.76) | (2.21,3.51) | (0.014) |
|  |  |  |  |  |  |  |  |  |  |  |  |
| **Age≥60 (n=284,848)** | | | | | | | | | | | |
| N of cases (prevalence %) | 33 (1.7) | 297 (2.5) | 1660 (4.1) | 4384 (6.1) | 6280 (7.7) | 3896 (8.1) | 1872 (9.2) | 634 (9.5) | 187 (10.6) | 114 (11.3) |  |
| OR ^a^ | 0.46 | 0.46 | 0.55 | 0.81 | 1.00 | 1.16 | 1.33 | 1.37 | 1.69 | 1.83 | 0.074* |
| (95% CI) | (0.20,1.04) | (0.27,0.77) | (0.41,0.74) | (0.70,0.93) | (reference) | (1.07,1.26) | (1.19,1.50) | (1.18,1.60) | (1.41,2.02) | (1.50,2.23) | (0.012) |
|  |  |  |  |  |  |  |  |  |  |  |  |

^a^ Meta-analysis estimates of cohort-specific OR adjusted for age and sex.

* P-value of difference across strata <0.001

OR, odds ratio; CI, confidence interval; SE, standard error
