# Supplementary material for: Body Mass Index and Diabetes in Asia: A Cross-Sectional Pooled Analysis of 900,000 Individuals in the Asia Cohort Consortium
Source: PLoS One. 2011 Jun 22;6(6):e19930. doi: 10.1371/journal.pone.0019930 (PMC3120751; doi:10.1371/journal.pone.0019930)
Supplement: References S1 — (DOCX) [file pone.0019930.s003.docx]

**Appendix References**

[a1] Marugame T, Sobue T, Satoh H, Komatsu S, Nishino Y, et al. (2005) Lung cancer death rates by smoking status: comparison of the Three-Prefecture Cohort study in Japan to the Cancer Prevention Study II in the USA. Cancer Sci 96: 120-6.

[a2] Matsuo T, Sairenchi T, Iso H, Irie F, Tanaka K, et al. (2008) Age- and gender-specific BMI in terms of the lowest mortality in Japanese general population. Obesity 16: 2348-55.

[a3] Tamakoshi A, Yoshimura T, Inaba Y, Ito Y, Watanabe Y, et al. (2005). Profile of the JACC study. J Epidemiol 15 Suppl 1:S4-8.

[a4] Tsugane S, Sobue T (2001) Baseline survey of JPHC study—design and participation rate. Japan Public Health Center-based Prospective Study on Cancer and Cardiovascular Diseases. J Epidemiol 11: S24-9.

[a5] Tsuji I, Nishino Y, Tsubono Y, Suzuki Y, Hozawa A, et al. (2004) Follow-up and mortality profiles in the Miyagi Cohort Study. J Epidemiol 14 Suppl 1:S2-6.

[a6] Tsuji I, Nishino Y, Ohkubo T, Kuwahara A, Ogawa K, et al. (1998) A prospective cohort study on National Health Insurance beneficiaries in Ohsaki, Miyagi Prefecture, Japan: study design, profiles of the subjects and medical cost during the first year. J Epidemiol 8: 258-63.

[a7] He J, Gu D, Chen J, Wu X, Kelly TN, et al. (2009) Premature deaths attributable to blood pressure in China: a prospective cohort study. Lancet 374: 1765-72.

[a8] Moy KA, Yuan J-M, Chung F-L, Wang X-L, Van Den Berg D, et al. (2009) Isothiocyanates, glutathione S-transferase M1 and T1 polymorphisms and gastric cancer risk: a prospective study of men in Shanghai, China. Int J Cancer 125: 2652-9.

[a9] Cai H, Yang G, Xiang YB, Hebert J, Liu D, et al. (2005) Sources of variation in nutrient intakes among men in Shanghai, China. Public Health Nutr 8: 1293-9.

[a10] Zheng W, Chow WH, Yang G, Fan J, Rothman N, et al. (2005) The Shanghai Women’s Health Study: rationale, study design, and baseline characteristics. Am J Epidemiol 162: 1123-31.

[a11] Chen CJ, You SL, Lin YP, CBCSP Study Group (1993) Community-based cancer screening project (CBCSP) in Taiwan. In: Chin BC, Yao C (eds.) Proceedings of 1993 Chinese American Academic and Professional Convention. Chinese American Academic and Professional Society, Oak Brook, pp. 937-40.

[a12] Chuang SY, Bai CH, Chen WH, Lien LM, Pan WH (2009) Fibrinogen independently predicts the development of ischemic stroke in a Taiwanese population: CVDFACTS study. Stroke 40: 1578-84.

[a13] Cho LY, Kim CS, Li L, Yang JJ, Park B, et al. (2009) Validation of self-reported cancer incidence at follow-up in a prospective cohort study. Ann Epidemiol 19: 644-6.

[a14] Hankin JH, Stram DO, Arakawa K, Park S, Low SH, et al. (2001) Singapore Chinese Health Study: development, validation, and calibration of the quantitative food frequency questionnaire. Nutr Cancer 39: 187-95.

[a15] Ahsan H, Chen Y, Parvez F, Hussain AZMI, Momotai H, et al. (2006) Health Effects of Arsenic Longitudinal Study (HEALS):  Description of a Multidisciplinary Epidemiologic Investigation.  J Exp Sci Environ Epidemiol 16: 191-205.

[a16] Pednekar MS, Hakama M, Hebert JR, Gupta PC (2008) Association of body mass index with all-cause and cause-specific mortality: findings from a prospective cohort study in Mumbai (Bombay), India. Int J Epidemiol 37: 524-35.
